# The protective effect of Epimedii Folium and Curculiginis Rhizoma on Alzheimer’s disease by the inhibitions of NF-κB/MAPK pathway and NLRP3 inflammasome

**DOI:** 10.18632/oncotarget.12574

**Published:** 2017-05-23

**Authors:** Zhou Lan, Guangjing Xie, Meng Wei, Ping Wang, Lvyi Chen

**Affiliations:** ^1^ School of Pharmacy, Hubei University of Chinese Medicine, Wuhan, P. R. China; ^2^ School of Basic Medicine, Hubei University of Chinese Medicine, Wuhan, P. R. China; ^3^ School of Pharmaceutical Sciences, South-Central University for Nationalities, Wuhan, P. R. China

**Keywords:** the water extracts of Epimedii Folium and Curculiginis Rhizoma (EX), Alzheimer’s disease, amyloid β, neuroinflammation, Gerotarget

## Abstract

The purpose of the current study was to explore the effects of the water extracts of Epimedii Folium and Curculiginis Rhizoma (EX) on Aβ-induced Alzheimer’s disease. Aβ_1-42_ was stereotaxically injected bilaterally into the dorsal hippocampus, and then the rats were orally received EX at the doses of 2 g/kg and 6 g/kg for 30 days. Behavior was monitored through Morris water maze test. The neuroprotective effect of EX were examined with methods of histochemistry and biochemistry. EX reduced the contents of pro-inflammatory cytokines tumor necrosis factor-alpha (TNF-α), interleukin-1 beta (IL-1β) and interleukin-6 (IL-6) in hippocampus and cortex. EX also reduced the levels of malondialdehyde (MDA) and increased superoxide dismutase (SOD), catalase (CAT), glutathione (GSH) and glutathione peroxidase (GSH-Px) in the serum. Immunohistochemical analysis demonstrated that EX inhibited the expressions of NLRP3. In addition, we further confirmed that EX suppressed the expression of the NLRP3 inflammasome. EX inhibited the phosphorylations MAPKs, nuclear factor κB (NF-κB), myeloid differentiation factor 88(MyD88), cathepsin B. In conclusion, these results suggest that EX may be a potential agent for treating Alzheimer’s disease.

## INTRODUCTION

Alzheimer's disease (AD), a common form of dementia, is a common neurogenerative disorder characterized by extracellular deposition of A beta (Aβ) plaques and intracellular accumulation of hyper-phosphorylated tau protein and NFTs (neurofibrillary tangles) [1]. The progressive accumulation of Aβ into oligomers and plaques and the inter-neuronal NFTs contribute to synaptic loss, neuronal dysfunction and death within vulnerable brain regions, leading to the progressive loss of memory and executive functions [2]. Currently, there are more than 38 million AD patients in the worldwide and every four seconds a new patient is diagnosed with AD. This number is expected to double every 20 years and to reach 115 million in 2050 due to the increasing aging population [3]. By the end of 2014, the number of Chinese aged 60 or older reached 212 million. The number of aging people is predicted to swell to more than one-third of the population by 2050, when China will become one of the countries most heavily burdened by AD [4]. Therefore, the effective treatment of AD becomes more urgent and remains a challenge.

At present, it is well accepted that AD is marked by neuroinflammatory events mediated by the resident neuroimmune cells of the brain, involving microglia and astrocytes [5]. One of the key pro-inflammatory cytokines involved in the neuroinflammatory process is interleukin-1β (IL-1β) [6]. IL-1β released from the plaque-surrounding microglia after stimulation with Aβ may contribute to deficits in long-term memory and neuronal dysfunction by promoting the formation of dystrophic neurite and its direct neurotoxicity [7]. The production and secretion of IL-1β require signals mediated by Toll-like receptors (TLRs) and NOD-like receptors (NLRs) [8]. On one hand, TLRs leads to the transcriptional induction of the pro-IL-1β *via* the activation of nuclear factor-κB (NF-κB) [9]. On the other hand, Aβ can active the NLRP3 inflammasome, a complex containing the apoptosis-associated speck-like protein (ASC), the protease caspase-1 and NLRP3, in microglia, which is fundamental for the secretion of IL-1β. IL-1β is primarily produced by pro-IL-1β to become biologically active. This process is mediated by the NLRP3 inflammasome which induces the activation of pro-caspase-1 to promote the maturation of pro-IL-1β [10]. These findings suggest that inflammation is proposed as a vital effector of AD.

To date, the anti-AD drugs mainly include acetylcholinesterase inhibitors, such as donepezil, rivastigmine and galantamine, and N-methylD-aspartic acid (NMDA) glutamate receptor antagonists (memantine) [11]. These therapeutic interventions for AD, however, have been primarily limited to treating symptoms without the ability to target the protection of the neurons. Once the intervention is interrupted, the symptoms of AD will be recovered. Consequently, there has been a great demand for new anti-AD agents capable of acting on multiple cytokines or mediators of inflammation. Fortunately, the effective treatments for AD are available from practitioners of traditional Chinese medicine. Traditional Chinese medicines exert their pharmacological effects through a multi-component and multi-target way in addition to their fewer side-effects, which provide the advantages and wide application possibilities compared with the pure drug with limited efficacy and some side effects. Therefore, researches have aimed at developing potent AD's drugs from Chinese medicines.

Epimedii Folium is the dried leaf of *Epimedium brevicornu* Maxim. and other 3 species in the genus Epimedium of the family Berberidaceae. It is warm in nature and acrid and sweet in taste. It can replenish *Shen-yang*, strong bones and muscles, and dispel wind and dampness. It has been commonly used for the treatment of atherosclerosis and neuropathy in traditional Chinese medicine. Moreover, Epimedii Folium has been proven to possess a wide range of efficacy including anti-inflammatory, anti-oxidant, anti-tumor and neuro-protective effects, etc. Pharmacological studies have also showed that icariin, a natural flavonoid compound extracted from Epimedii Folium, can remarkably attenuate cognitive deficits in several different AD's models [12–13].

Curculiginis Rhizoma is the dried rhizoma of *Curculigo orchioides* Gaertn. (It belongs to the family Amarylidaceae). *Curculigo orchioides* Gaertn. is a small herb widely distributed in China, Malaya, Japan, India and Australia. It has the properties of invigorating *Shen-yang*, expelling cold and eliminating dampness. It has been commonly used to treat impotence, cold sperm and aging diseases as a traditional Chinese medicine in China for a long time. In addition, recent studies have manifested that compounds isolated from Curculiginis Rhizoma had a wide spectrum of pharmacological activities, including neuro-protective, anti-oxidant, anti-inflammatory, anti-bacterial, anti-osteoporotic and estrogenic effects, etc [14–15].

In China, Epimedii Folium and Curculiginis Rhizoma are often used together as herb pairs in formula on clinic in order to obtain a synergistic effect for reinforcing *Shen-yang* and relieving pain to treat aging diseases. For example, the famous traditional Chinese medicine formula Er-Xian decoction containing Epimedii Folium and Curculiginis Rhizoma as the main herbs has been used for the treatment of osteoporosis disorders, menopausal syndrome and aging diseases for several decades [16]. In this formula, Epimedii Folium and Curculiginis Rhizoma are succinctly combined with the therapeutic merit of warming *Shen-yang*. Although Epimedii Folium and Curculiginis Rhizoma are excellently combined as herb pair on clinic, the evaluation of effects and mechanisms of this herb pair are lack. A number of researches have more focused on the pharmacological activities of Epimedii Folium and Curculiginis Rhizoma used alone other than together. Hence, the mechanisms of the combined extracts of Epimedii Folium and Curculiginis Rhizoma were investigated in Aβ_1-42_-induced AD rats in this study.

## RESULTS

### TOF-MS analysis of EX

**Figure 1:**
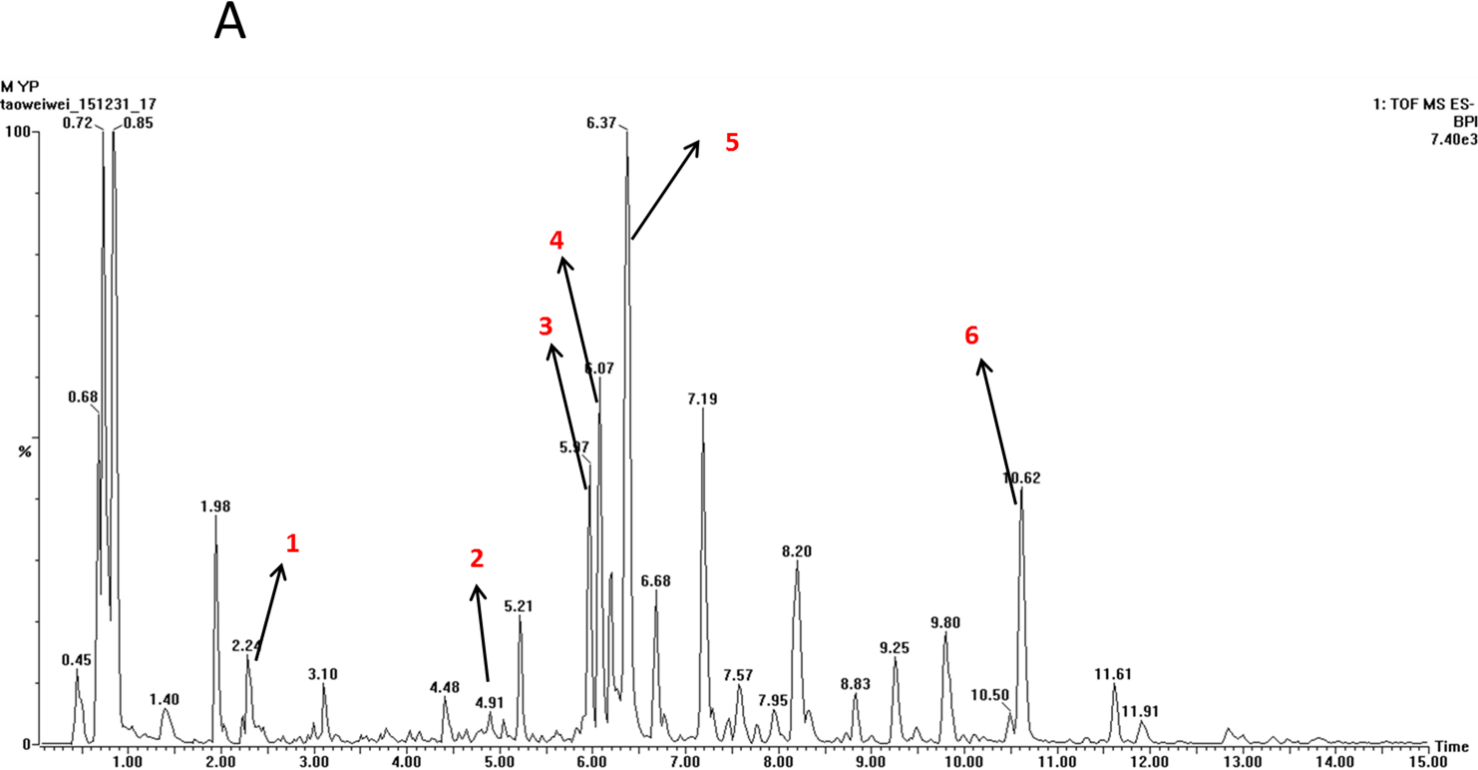

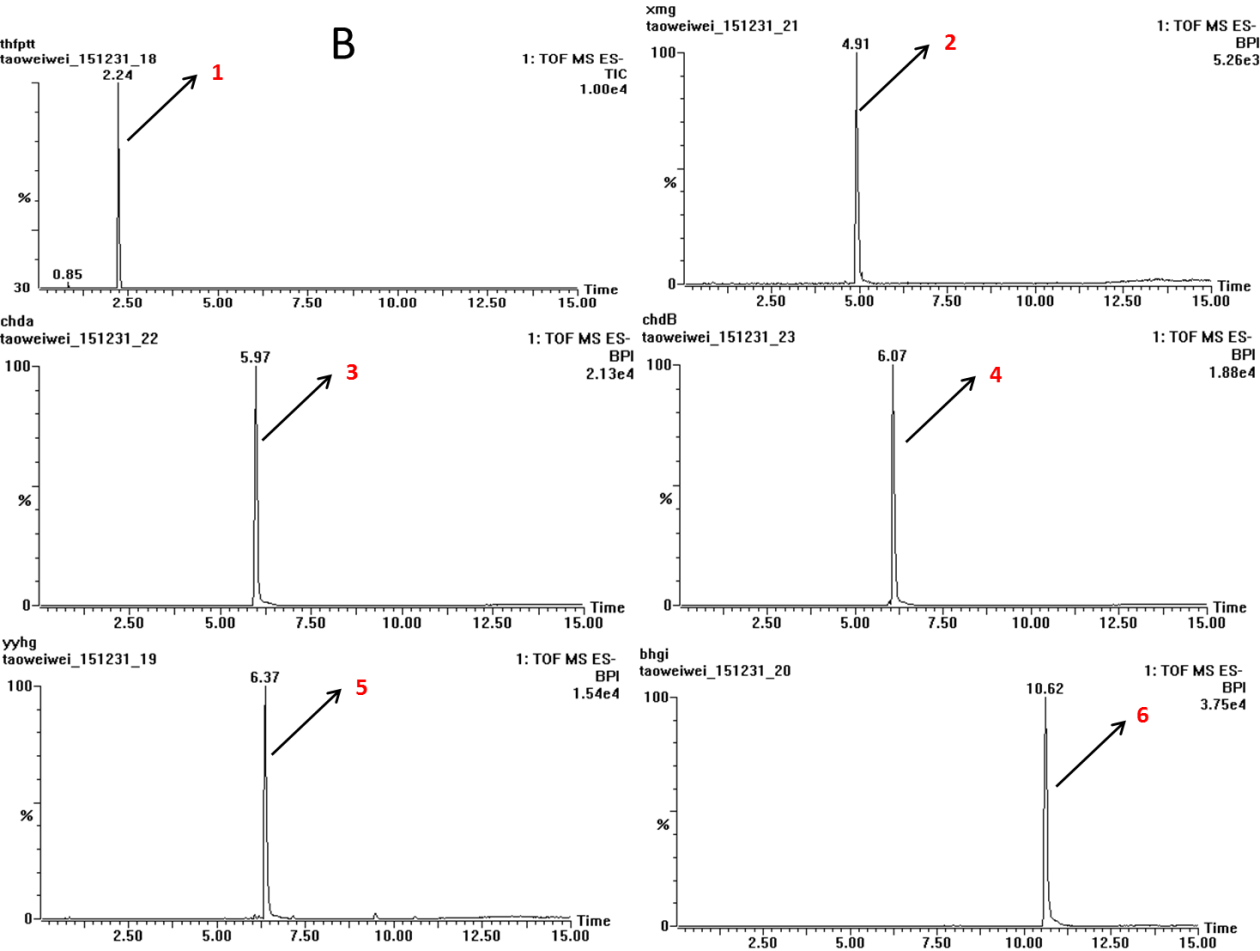

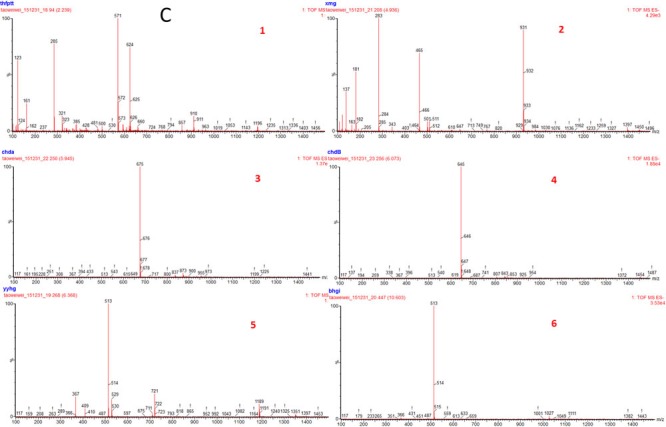
TOF-MS chromatogram of EX (1) Orcinol glucosid; (2) Curculigoside; (3) Epimedin A; (4) Epimedin B; (5) Icariin; (6) Baohuoside I.

The typical TOF-MS chromatogram of EX was shown in Figure 1. By comparing both the retention times of the reference standards and the spectra of MS, six compounds, including Orcinol glucosid (1), Curculigoside (2), Epimedin A (3), Epimedin B (4), Icariin (5), Baohuoside I (6) were well indentified.

### Effect of EX on spatial learning and memory ability

**Figure 2:**
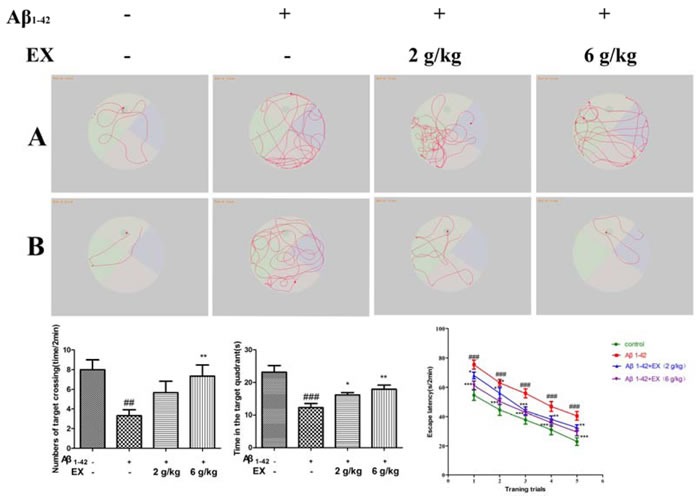
The effect of EX on the performance in Morris water maze of Aβ_1-42_-induced rats **A.** Representative searching strategy of rats in the second trial on the second day. **B.** Representative searching strategy of rats in the second trial on the fifth day. **C.** Numbers of target crossing of the rats in all groups. **D.** The time of the rats spent in the quadrant where the platform was once placed within 120s. E. Escape latency to find the hidden platform during the five consecutive days training. Data are expressed as mean ± S.E.M., *n* = 8. ^#^*p* < 0.05 *versus* the control; * *p* < 0.05 *versus* the Aβ_1-42_-treated group. Data were analyzed with ANOVA followed by Duncan's Multiple Range Test.

The spatial learning and memory ability of animals was assessed by Morris water maze test. Figure 2 showed that the mean latency to find the platform declined progressively during the five training days. The Aβ_1-42_-treated rats remarkably spent longer time on finding the platform than the control ones did on the second day onwards (*p* < 0.05). These results revealed that the Aβ_1-42_-treated rats had obvious cognitive impairment. Moreover, the increase of escape latency was shortened respectively by EX 2 g/kg from the third to fourth days (*p* < 0.01 *vs*. the Aβ_1-42_-treated model rats) and by EX 6 g/kg from the first to fifth days (*p* < 0.05 *vs*. Aβ_1-42_-treated model rats). Figure 2 illustrated the swim paths of rats in the second trial on the second and the fifth days in this test. Rats tended to explore all four quadrants of the pool in the second day. On the fifth day, the control rats swam in the direction of the platform, however, Aβ_1-42_-treated rats took longer swimming paths. In the probe test (Figure 2), the control rats spent almost twice more of the time in the quadrant where the platform was once placed than the Aβ_1-42_-treated model rats (*p* < 0.05). Besides, compared with the Aβ_1-42_-treated model rats, EX (2 and 6 g/kg) treated rats took longer time in the target quadrant (*p* < 0.05, respectively).

### Effects of EX on pro-inflammatory cytokines (IL-1β, IL-6 and TNF-α)

**Figure 3:**
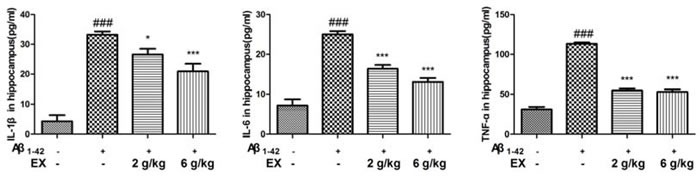
Effects of EX on pro-inflammatory cytokines (IL-1β, IL-6 and TNF-α) in hippocampus of Aβ1-42-induced rats Data were expressed as mean ± S.E.M. (*n* = 8). ^##^*p* < 0.01 and ^###^*p* < 0.001 *versus* the control; * *p* < 0.05, ^*^ *p* < 0.01 and ^**^* *p* < 0.001 *versus* the Aβ_1-42_-treated group. Data were analyzed with ANOVA followed by Duncan's Multiple Range Test.

**Figure 4:**
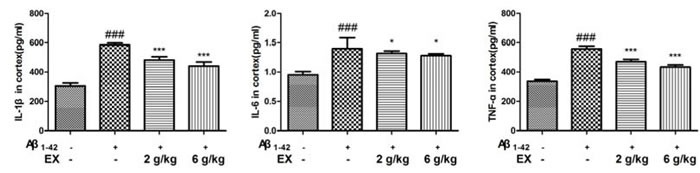
Effects of EX on pro-inflammatory cytokines (IL-1β, IL-6 and TNF-α) in cortex of Aβ1-42-induced rats Data were expressed as mean ± S.E.M. (*n* = 8). ^##^*p* < 0.01 and ^###^*p* < 0.001 *versus* the control; * *p* < 0.05, ^*^ *p* < 0.01 and ^**^* *p* < 0.001 *versus* the Aβ_1-42_-treated group. Data were analyzed with ANOVA followed by Duncan's Multiple Range Test.

To assess the impact of EX on the Aβ_1-42_-mediated inflammatory response, the ELISA kits were applied to determine the production of IL-1β, IL-6 and TNF-α. As shown in Figure 3-4, the elevated levels of IL-1β, IL-6 and TNF-α significantly were found in the mice which were subjected to intrahippocampal Aβ_1-42_ injection. However, EX treatments (2 g/kg, 6 g/kg) significantly declined the production of IL-1β, IL-6 and TNF-α in the hippocampus and cortex after intrahippocampal Aβ_1-42_ injection. These results demonstrated that EX might ameliorate Aβ_1-42_-induced overproduction of pro-inflammatory cytokines, which was evidenced by the reverse effect of EX on the increase in the levels of IL-1β, IL-6 and TNF-α.

### Effects of EX on oxidation-related indicators (SOD, MDA, GSH-Px and CAT)

**Figure 5:**
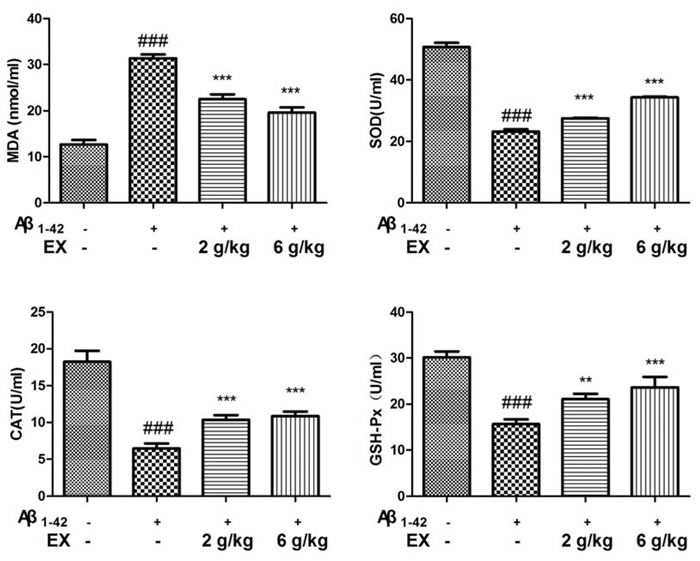
Effects of EX on the levels of SOD, MDA, CAT, GSH-Px in serum of Aβ 1-42-induced rats The data expressed as mean ± S.E.M (*n* = 8). ^#^*p* < 0.05, ^##^*p* < 0.01 and ^###^*p* < 0.001 *versus* the control; * *p* < 0.05, ^*^ *p* < 0.01 and ^**^* *p* < 0.001 *versus* the Aβ_1-42_-treated group.

To examine the anti-oxidative property of EX, the levels of MDA, SOD, CAT and GSH-Px in serum were detected. As depicted in Figure 5, Aβ_1-42_ injection was proved to significantly decline the SOD, CAT and GSH-Px activities with the increase of MDA content in the serum. On the contrary, treatment with EX (2, 6 g/kg) remarkably restored the activities of SOD, CAT and GSH-Px to different degree in the serum compared with those in model group. Additionally, an elevation was exhibited in the MDA content after EX (2, 6g/kg) treatment with a more apparent increase of MDA at the dose of EX (6 g/kg), as compared with the model group of merely Aβ_1-42_ injection.

### Effects of EX on NLRP3 inflammasome activation

**Figure 6:**
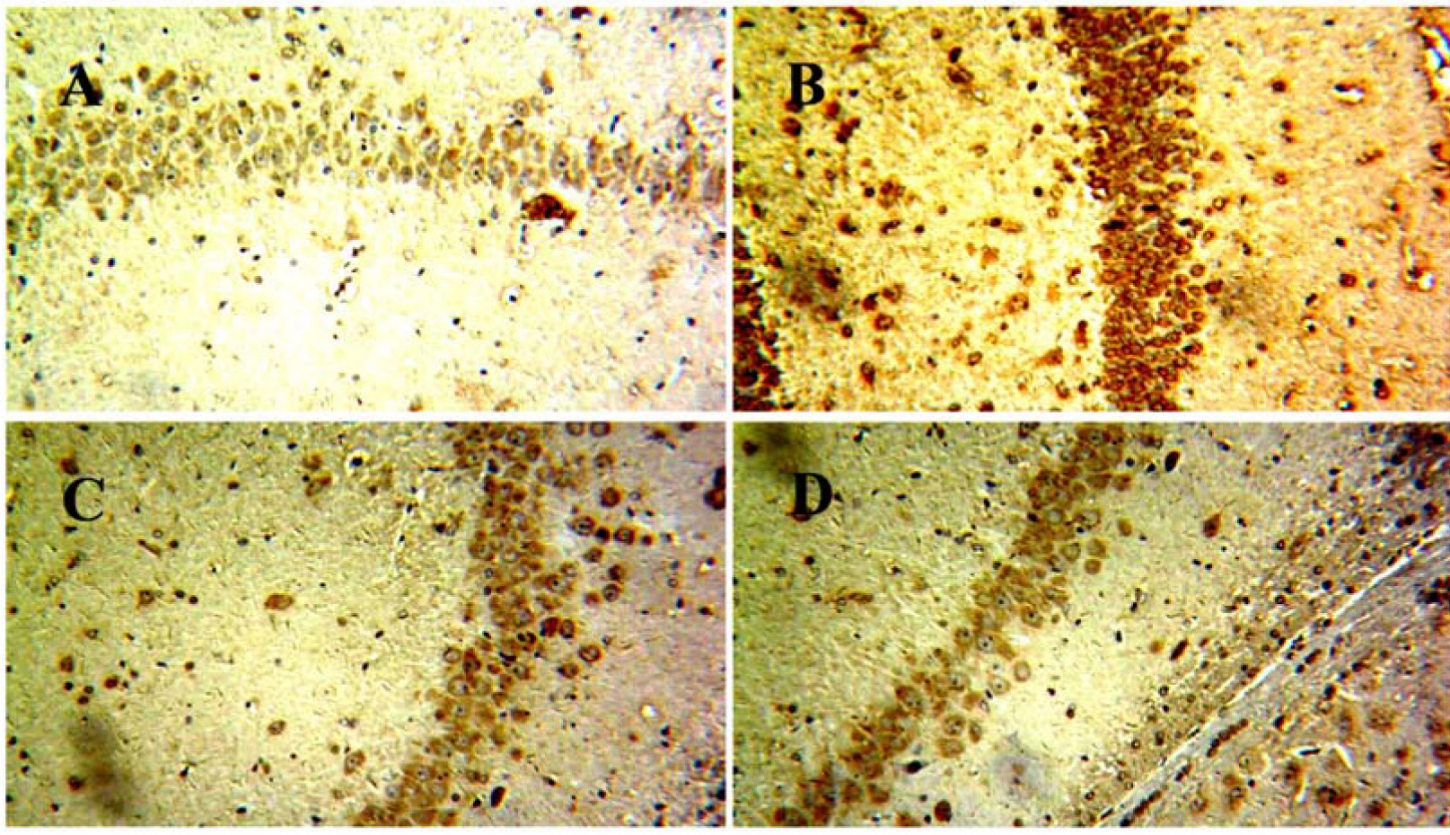
The effects of EX on the expression of NLRP3 in brain of Aβ 1-42-induced rats by immunohistochemical analysis **A.** Contol; **B.** Model; **C.** EX(2g/kg); **D.** EX(6g/kg).

**Figure 7:**
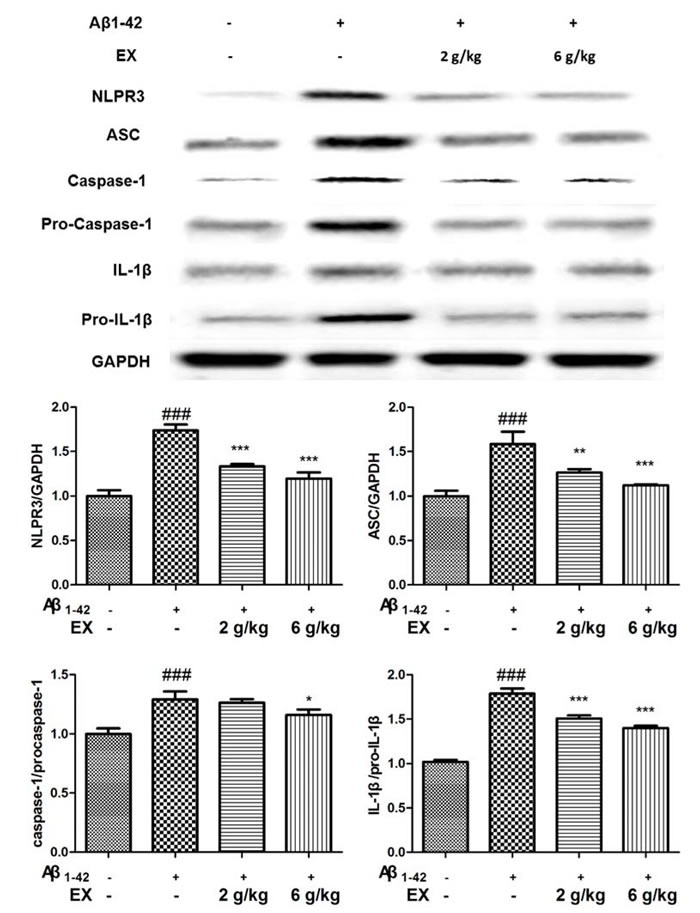
Effects of EX on the activation of NLRP3 inflammasome-related protein of Aβ _1-42_-induced rats The data expressed as mean ± S.E.M (*n* = 3). ^#^*p* < 0.05, ^##^*p* < 0.01 and ^###^*p* < 0.001 *versus* the control; * *p* < 0.05, ^*^ *p* < 0.01 and ^**^* *p* < 0.001 *versus* the Aβ_1-42_-treated group.

**Figure 8:**
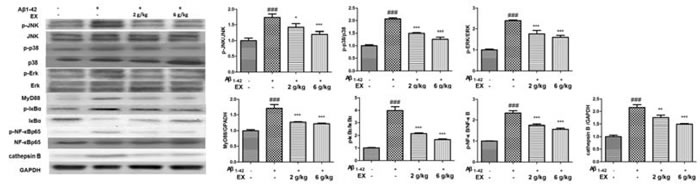
Effects of EX on the activation of MAPK/NF-κB pathway-related protein of Aβ 1-42-induced rats The data expressed as mean ± S.E.M (*n* = 3). ^#^*p* < 0.05, ^##^*p* < 0.01 and ^###^*p* < 0.001 *versus* the control; * *p* < 0.05, ^*^ *p* < 0.01 and ^**^* *p* < 0.001 *versus* the Aβ_1-42_-treated group.

Mounting evidence had highlighted that NLRP3 activation which was related to Aβ degradation played an essential role in the pathological progression of AD [17]. Immunohistochemical staining showed that EX could attenuated the expressions of NLRP3 in brains (Figure 6). Accordingly, we investigated the effect of EX on NLRP3 inflammasome activation. The data depicted in Figure 7 illustrated the highly expressed NLRP3, ASC, Caspase-1, and IL-1β in model group, while the administration of EX (2 g/kg, 6 g/kg) and dramatically inhibited the expressions of them to varying degrees with GAPDH. Results obtained from western blots (Figure 8) suggested the increased expression of MAPK- NF-κB axis in model rats and decreased of them in EX (2 g/kg, 6 g/kg) treated groups, which further confirmed the involvement of inflammation pathway in Aβ_1-42_ -induced AD.

## DISCUSSION

In the present study, we investigated the effect of EX on cognitive function and neuroinflammatory in Aβ-treated mice. Aβ_1-42_ is reported to be generated by successive cleavage of the amyloid precursor protein (APP) through β-secretase and γ-secretase [18], which was employed to induce AD to verify the effects of EX. And the AD model was successfully developed by the assessment of Morris water maze test (MWM), which was in line with the findings from prior works [19]. On the one hand, poor performance was observed in the MWM test in Aβ_1-42_-induced cognitive dysfunction, which was in agreement with previous literatures [20]. On the other hand, EX considerably declined the escape latency of AD rats in the MWM test, implying that EX could enhance hippocampal-mediated learning and memory abilities in AD rats.

Previous research has demonstrated that inflammation plays a vital role in the progression of poor cognitive performance [21]. It was suggested that inflammation was one of the mechanisms related to AD characterized by progressive memory disorders and cognitive dysfunction [22]. The released cytokines, in particular IL-1β, IL-6 and TNF-α, are the major effectors of the neuroinflammatory signals, and can affect neurophysiologic mechanisms regarding cognition and memory [23–24]. It is well known that systemic pro-inflammatory cytokines could enter CNS by invading in the impaired blood brain barrier (BBB), accelerating the following occurrence of neurodegeneration [25]. Hence, pro-inflammatory cytokines TNF-α, IL-6 and IL-1β in hippocampus were measured to evaluate the severity of inflammation injury in the brain of AD rats [26]. The acquired data suggested that EX might attenuate the defect of memory and learning capability through the inhibition of pro-inflammatory cytokines.

Three major upstream mechanisms related NLRP3 activation have been proposed: reactive oxygen species (ROS), ion fluxes, or phagosomal destabilization [27]. Therefore, we focused on the ROS, the origin of oxidative stress by examination the generation of oxidative stress markers (MDA, SOD, CAT and GSH-Px) [28]. Varying degree of decrease in the MDA content were observed in the serum in Aβ_1-42_-induced rats treated with EX (2 g/kg, 6 g/kg), compared with those in model mice stimulated by Aβ_1-42_. We also examined the effects of EX on the enzymatic activities of intracellular antioxidant enzymes such as SOD, CAT and GSH-Px in the serum. Increased SOD, CAT and GSH-Px activities inhibited by Aβ_1-42_ injection was observed in the AD rats treated with EX (2 g/kg, 6 g/kg). Our results above implied that the anti-oxidative ability of EX might contribute to alleviate the development of AD.

Previous investigators revealed that the degradation of extracellular Aβ fibrils by microglia is dependent on autophagic processes [17]. Autophagy is also important for the regulation of Aβ-mediated NLRP3 inflammasome activation, thereby affecting neuronal survival. The extracellular accumulation of β-amyloid (Aβ), regarded as the primary causative factor resulting in neurodegeneration, was thought to be one of the irrefutable pathological findings of AD [29]. Sufficient studies points toward a dysregulation of the immune system and inflammation as potential switch for onset AD. Activated microglia in senile plaques has pro-inflammatory phenotype linked with neurotoxicity [30]. Moreover, Aβ fibrils activate the NLRP3 inflammasome resulting in release of pro-inflammatory cytokine IL-1β in microglia and NLRP3 inflammasome is activated in the brains of AD patients [31]. The results of western blot revealed that the high expressions of NLRP3/ASC/IL-1β pathway-related proteins in AD rats were effectively inhibited by EX (2 g/kg, 6 g/kg) to different degrees, indicating the impact of NLRP3 on AD and the effectiveness of EX in the treatment of AD rats induced by Aβ_1-42_ administration.

To further identify the molecular mechanism related to the recovery ability of the brain of AD rats by EX treatment, we utilized the western blot to clarify the relationship between the relative expressions of MAPK and NF-κB pathway in AD rats. Former literature proposed the involvement of MAPK and NF-κB signaling in MAPK and NF-κB [32]. The closely relationship between MAPK and NF-κB has been documented [33]. A growing number of links have put forward a strong correlation between MAPK signaling pathway and AD. Aβ increased the activation of JNK, which is involved in the following injury and death [34]. Mohammadi et al. indicated JNK inhibitor performed protection against Aβ through the declined of autophagy and then alleviated memory deficit stimulated by Aβ [35]. Meantime, it is well documented that NF-κB is a major regulator for the generation of inflammatory cytokines [36] Upon inflammation stimulation, IκBα is phosphorylated at serine residues and degraded rapidly following IKK complex activation [37]. The phosphorylation of p65 subunit is also promoted, and then p65 phosphorylation enhances transcriptional activity of NF-κB followed by the regulation of pro-inflammatory cytokine production [38]. The data of our study was consistent with previous studies, suggesting that EX administration lowered Aβ_1-42_-induced the expression of MAPK related protein and suppressed MAPK-mediated inflammatory response as evidenced by diminished levels of NF-κB p65, TNF-α and IL-1β in the hippocampus.

Taken together, our finding described the pharmacological effect of EX on AD. Both inflammation and oxidative stress were considered as the vital factors affecting the function of brain. Besides, the involvement of NLRP3 inflammasome activation might lead to new insights into the underlying pathophysiology of cognition impairment.

## MATERIALS AND METHODS

### Animals

Adult male Sprague-Dawley rats (250~300 g, Comparative Medical Center of Yangzhou University, Yangzhou, China) were housed one week to adapt to their environment before used for experiments. They were maintained on standard laboratory conditions of temperature 25 ± 1 °C and a 12-h light/12-h dark cycle with food and water available *ad libitum* for the duration of the study. All the experiments and animal care were performed strictly in accordance with the Provision and General Recommendation of Chinese Experimental Animals Administration Legislation and were approved by the Science and Technology Department of Jiangsu Province.

### Materials and chemicals

Epimedii Folium and Curculiginis Rhizoma were purchased from Tongrentang drugstore in Wuhan, Hubei province (China). They were authenticated by the corresponding author. The voucher specimens were deposited in the Department of Pharmacognosy, Hubei University of Chinese Medicine. Orcinol glucosid, Curculigoside, Epimedin A, Epimedin B, Icariin and Baohuoside I were purchased from the National Institute for the Control of Pharmaceutical and Biological Products (Beijing, China). The purity of each compound was determined to be higher than 95% by HPLC. Each reference compound was accurately weighed and dissolved in methanol as stock solutions. Kits used for IL-1β, IL-6 and tumor necrosis factor-α (TNF-α) enzyme linked immunosorbent assay (ELISA) were purchased from Shanghai Westang Bio-tech CO., Ltd., Shanghai, China. Kits for superoxide dismutase (SOD), melonydialdehyde (MDA), and catalase (CAT) were purchased from Nanjing Jiancheng Institute of Biological Engineering (Nanjing, China). Other reagents were AR grade.

Epimedii Folium and Curculiginis Rhizoma were mixed in a ratio of 1:1. The mixture was extracted twice for 2h each time by distilled water (1:10, w/v). The filtrates were concentrated and dried in vacuum at 60 °C, and then stored at 4 °C. The yield of dried powder was 15.5% according to the original dry material. The doses of water extracts of Epimedii Folium and Curculiginis Rhizoma (EX) were expressed as gram of the original dry materials per kilogram body weight.

### TOF-MS analysis of EX

The multi-components of EX were characterized by TOF-MS. The sample was analyzed using Thermo Syncronic C18 column (100 mm × 2.1 mm, 1.7 μm) with the detector wavelength set at 254 nm. The mobile phase consisted of CH_3_CN (A) and 0.1% (v/v) CH_3_COOH (B). A gradient program was used as follows: 0-3 min, 5% A-95% B; 3-9 min, 30% A-70% B; 9-12 min, 65% A-35% B; 12-14 min, 95% A-5% B; 14-15 min, 5% A-95% B. The flow rate was 0.4 mL/min.

### Animal treatment paradigm

The soluble Aβ_1-42_ (Sigma-Aldrich, St. Louis, MO, USA) was dissolved in filtered phosphate buffered saline (PBS, pH 7.5, 0.1M) at a concentration of 200 μg/μL, and then incubated at 37 °C for seven days. On the test day, the solution was diluted with PBS to reach the final concentration of 1 μg/μL.

Rats were anaesthetized by intraperitoneal injection of chloral hydrate (320 mg/kg), and placed into stereotaxic device. After cleaning the area surrounding bregma, 5 μL aggregated Aβ_1-42_ (1 μg/μL) or PBS were stereotaxically injected bilaterally into the dorsal hippocampus at a rate of 0.5 μL/min. The injected position was coordinated relative to the bregma: - 3.8 mm posterior to bregma, ± 2.5 mm lateral to midline and - 3.0 mm to the dura ^39^. The rats were left in a temperature-controlled chamber until they recovered from anesthesia. Penicillin-G 200,000 IU/mL was administered after surgery (0.2~0.3 mL/rat, intramuscular).

One day after the operation, the Aβ_1-42_-treated rats were randomly divided into 3 groups as follows: group 2 (*n* = 8) that served as model group received the PBS (p.o.); group 3 (*n* = 8) and 4 (*n* = 8) received EX at the doses of 2 g/kg and 6 g/kg (p.o.), respectively. Meanwhile, PBS-intrahippocampal injected rats served as control group (*n* = 8) received the PBS (p.o.). The drugs were orally administered through feeding tube daily for 30 consecutive days.

### Morris water maze test

Spatial memory ability was detected by Morris water maze test [40] with minor modifications. The Morris water maze, which consisted of 5-6 days place navigation training and a probe test on the day 6, was carried out in a black circular pool (180 cm in diameter and 60 cm in height) with a featureless inner surface. A round escape platform was placed in 1 cm underneath the water surface in the center of one quadrant. The rats were given two trial sessions each day for five consecutive days, with an inter-trial interval of 20 min, and the escape latencies were recorded. Once the rat located the platform, it was permitted to remain on it for 10 s. If the rat did not locate the platform within 120 s, it was placed on the platform for 15 s and the escape latency was recorded as 120 s. On day 6, the platform was removed and each rat was allowed to swim freely for 120 s as the probe test. The time that rat spent in the target quadrant (where the platform was once hidden) was measured.

### Preparation of tissue samples

Rats were decapitated 60 min after the behavioral tests. Brains were removed carefully and quickly to 0.9% cold saline and the hippocampus were immediately dissected on a cold plate, weighed and homogenized with ice-cold saline. The homogenate was centrifuged at 3000 rpm for 10 min at 4 °C and the supernatant was used.

### Determination of cytokines (IL-1β, IL-6, TNF-α, MDA, SOD, CAT and GSH-Px)

Levels of IL-1β, IL-6 and TNF-α in hippocampus and cortex were determined by ELISA kits according to the manufacture instructions. Cytokine concentrations in the test samples were evaluated with reference to the standard curves prepared using recombinant cytokines of known concentrations.

Activities of MDA, SOD, CAT and GSH-Px in the serum were also measured by the methods described in the assay kits. SOD activities (U/ml) of samples were expressed with reference to the activity of a standard curve of bovine erythrocyte SOD dilutions. One unit was defined as the amount of enzyme needed to exhibit fifty percent dismutation of the superoxide radical. Absorbance values of both samples and standards were measured at 440 nm by a micro-plate reader. The amounts of TBARS such as MDA (nmol/ml) in samples were measured by the reaction with TBA. CAT activities (U/ml) of samples were measured on the basis of the activity that catalyzed the conversion of H_2_O_2_ to oxygen and water. GSH-Px activities (U/ml) of samples were measured by the content of NADPH.

### Western blot analysis

The hippocampus was lysed in RIPA buffer containing protease or phosphatase inhibitors. Protein concentrations were determined by the Bradford protein assay. Protein lysates were separated by SDS-PAGE electrophoresis and were transferred onto polyvinylidene difluoride (PVDF) membranes. Antibodies for Western blots were from Cell Signaling Technology unless indicated. Products numbers and antibody dilutions are indicated within parentheses. After blocking with 5% BSA for 1 hour, the membranes were incubated at 4 °C overnight with the following antibodies: NLPR3 (#15101, 1:1000), ASC (#13833, 1:1000), caspase-1 (#2225, 1:1000), pro-caspase-1 (#2225, 1:1000), IL-1β (#12703, 1:1000), pro-IL-1β (#12703, 1:1000), NF-κB p65 (#3034, 1:1000), phospho-NF-κB p65 (#3031, 1:1000), JNK (#4672, 1:1000), p-JNK (#4668, 1:1000), P38 (#8690, 1:1000), p-P38 (#4511, 1:1000), Erk (#4695, 1:1000), p-Erk (#4094, 1:1000), IκBα (#4766, 1:1000), p-IκBα (#4766, 1:1000), MyD88 (#4283, 1:1000), cathepsin B (Santa Cruz Biotechnology, sc-13985, 1:500), GAPDH (#5174, 1:1000). Subsequently, the membranes were incubated with the appropriate horseradish peroxidase-conjugated secondary antibody for 1 hour. Bands intensity was measured using the SuperSignal West Pico Chemiluminescent Substrate (Thermo Fisher Scientific Inc.) and normalized by corresponding loading control proteins.

### Statistical analysis

All values were expressed as the mean ± S.E.M. and analyzed by one-way analysis of variance (ANOVA) followed by Duncan's Multiple Range Test using SPSS version 13.0 software; a *p*-value of less than 0.05 was considered significant.

## Abbreviations

Aβ: β-amyloid peptide
AD: Alzheimer's disease
CAT: catalase
EX: the water extracts of Epimedii Folium and Curculiginis Rhizoma
IL-1β: interleukin-1β
MDA: melonydialdehyde
NF-κB: nuclear factor κB
SOD: superoxide dismutase
TNF-α: tumor necrosis factor-α.
